# Analysis of General Knowledge on Obstructive Sleep Apnea Syndrome (OSAS) among Italian Pediatricians

**DOI:** 10.3390/children11020148

**Published:** 2024-01-24

**Authors:** Stefano Saran, Sabina Saccomanno, Simonetta Viti, Rodolfo Francesco Mastrapasqua, Grazia Viti, Nicola Giannotta, Paola Fioretti, Elisa Lorenzini, Luca Raffaelli, Luca Levrini

**Affiliations:** 1 Department of Human Sciences, Innovation and Territory, School of Dentistry, Postgraduate of Orthodontics, University of Insubria, 21100 Varese, Italyngiannotta@studenti.uninsubria.it (N.G.); luca.levrini@uninsubria.it (L.L.); 2 Orthodontic Residency, Department of Life, Health and Environmental Sciences, University of L’Aquila, 67100 L’Aquila, Italy; sabinasaccomanno@hotmail.it; 3 Department of Dentistry, Dental School, IRCCS San Raffaele Hospital, Vita-Salute San Raffaele University, 20132 Milan, Italy; s.viti@studenti.unisr.it; 4 ENT Department, Rivoli Hospital, ASL TO3, 10098 Rivoli, Italy; rfmastrapasqua@aslto3.piemonte.it; 5 Department of Medicine and Surgery, Hygiene and Public Health Section, University of Perugia, 06123 Perugia, Italy; paola.fioretti2@studenti.unipg.it (P.F.); elisa.lorenzini1@studenti.unipg.it (E.L.); 6 Dental School, Catholic University of the Sacred Heart, 00168 Rome, Italy; luca.raffaelli@unicatt.it

**Keywords:** OSAS, pediatric, apnea, pediatricians, dentistry

## Abstract

Introduction: Obstructive sleep apnea syndrome (OSAS) is a disorder characterized by partial or total airway obstruction during sleep. Studies have shown variability in the level of knowledge and awareness about OSAS among pediatricians. The management of childhood obstructive sleep apnea syndrome (OSAS) depends on the severity of the disease, the presence of comorbidities, and the child’s age. The American Pediatric Academy recommends a multidisciplinary approach involving a pediatrician, a sleep specialist, and an otolaryngologist to provide comprehensive care for children with OSAS. The aim of this cross-sectional study is to evaluate the level of knowledge among pediatricians in Italy regarding the diagnosis of pediatric OSAS. Material and Methods: An anonymized survey was conducted among Italian pediatricians. The survey was administered electronically using Google Forms, and a total of 350 pediatricians were invited to participate. Out of the 350 invitations, 299 pediatricians responded to the survey. The statistical analysis performed consisted of descriptive analysis. The study included 297 pediatricians. Results: Pediatricians demonstrated proficiency in identifying common nocturnal and day symptoms of OSAS. A majority (68.9%) considered the oral and otorhinolaryngologist areas during checkups. Approximately 70.6% took patient weight into account, and 62.8% were aware of the regional diagnostic-therapeutic-assistance pathway. Conclusions: According to the results of this manuscript, there is evidence of a good level of knowledge about OSAS, but disseminating more information about OSAS and all the health issues associated with this syndrome is suggested. This study also has limitations caused by the complexity of the pathology.

## 1. Introduction

Obstructive sleep apnea syndrome (OSAS) is a disorder characterized by partial or total upper airway obstruction during sleep. While it primarily affects adults, it can also be diagnosed in children and is associated with various systemic issues. There is a clear relationship between altered sleep patterns and the development of OSAS [1].

Furthermore, OSAS is connected to cardiovascular disease, increased insulin resistance, and high blood pressure [2]. Current literature highlights a strong link between poor school performance and OSAS in children [1]. Additionally, an alteration of the Insulin-like growth factor (IGF-I) axis factor is linked to this syndrome [3]. The IGF-I axis is the principle endocrine system regulating linear growth in children.

The typical signs and symptoms of OSAS in childhood are snoring, difficulty breathing during sleep, hypercapnia, hypoxemia, sleep fragmentation, and rates of apnea and hypopnea higher than 1 per hour, as well as enuresis [3,4,5]. According to some authors, this syndrome can affect cognitive function and can influence the psychophysical growth of children. Polysomnographic analysis is used to diagnose this disease, which is important since some of the signs and symptoms are subtle, and parents may not be aware of this syndrome in most cases [4].

The pathophysiology of OSAS is complex and likely multifactorial [6]. The incidence rate of the disease among the pediatric population is between 2 and 4%, particularly among children between 2 and 8 years old [7]. OSAS is increasing among children affected by infantile obesity, which, along with adenoids and tonsil hypertrophy, is the most common apparent cause of OSAS. If obesity is present, the first approach should be an attempt to reduce BMI [8,9]. Weight management is an essential part of the therapeutic approach for OSAS in children who are overweight or obese [10,11]. Other factors that seem to increase the risk of OSAS include allergic rhinitis, abnormal craniofacial morphologies, neurological problems, genetic disorders, hypothyroidism, familial prematurity, mucopolysaccharidosis, and familial predisposition for OSAS [12]. OSAS can be related to a backward-positioned profile, in which there is a reduced posterior airway space (PAS) [13].

It is important to note that the severity of the disease does not depend solely on the rate of hypopnea and apnea per hour, and some children can have more symptoms and a higher complexity of clinical involvement. Cases with a apnea–hypopnea index (AHI) > 5/h can have signs and symptoms depending on a more comprehensive analysis of the polysomnography and, mainly, the clinical case presented by the child. Cases with 1/h ≤ AHI ≤ 5/h have mild clinical complexity [14].

The management of childhood obstructive sleep apnea syndrome (OSAS) depends on the severity of the disease, the presence of comorbidities, and the child’s age. The American Pediatric Academy recommends a multidisciplinary approach involving a pediatrician, a sleep specialist, and an otolaryngologist to provide comprehensive care for children with OSAS [4,15].

The first-line treatment for mild to moderate OSAS is usually the removal of the tonsils and adenoids [16]. Continuous positive airway pressure (CPAP) therapy is recommended for children with severe OSAS [17]. It attempts to open up the lungs’ peripheral airways. It increases airway pressure, preventing the collapse of poorly supported peripheral small airways during expiration.

Orthodontic treatments and behavioral interventions may be helpful in improving the upper airway and reducing OSAS symptoms [18,19].

Pediatricians play a pivotal role in screening and diagnosis of OSAS because they are the first medical contact for pediatric patients, so they can intercept these kinds of diseases.

Some pediatricians have reported feeling confident in their knowledge and ability to diagnose and manage OSAS in children, while others have expressed less confidence and a need for further education and training on the topic. This variability in knowledge and awareness can have important implications for early detection and appropriate management of OSAS in children. A survey of pediatricians in the United States found that only 25% reported routinely screening for OSAS in their patients, and only 39% felt comfortable managing OSAS. Another study found that only 20% of pediatricians were able to correctly identify the criteria for diagnosing OSAS [17].

In summary, although pediatricians play a critical role in managing respiratory disorders in children, there is variability in their level of knowledge and confidence. Continued education and training can help improve their ability to diagnose and manage respiratory disorders and promote good respiratory health in children. The aim of this study is to evaluate the level of knowledge among pediatricians in Italy regarding the diagnosis of pediatric OSAS.

## 2. Material and Methods

### 2.1. Study Participants

An anonymized survey was conducted among Italian pediatricians working in public and private practices. The survey was administered electronically using Google Forms, and a total of 350 pediatricians were invited to participate through e-mail. Out of the 350 invitations, 299 pediatricians responded to the survey during the period that was from November 2022 to March 2023. Of the respondents, 155 were male, 142 were female, and 2 did not specify their sex. The pediatricians were selected in a homogeneous way from different areas of the country. 

The survey was conducted using Google LLC, located at 1600 Amphitheatre Parkway, Mountain View, California, U.S. An electronic platform was used for data collection and analysis. No reminders were sent to the clinicians to ensure their freedom to participate. The purpose of the questionnaire was clearly stated as seeking ways to improve patient care. To ensure data quality, it was ensured that each pediatrician provided only one response by checking for differences in answers and response timings. Pediatricians involved in the study accepted to answer the survey, and no financial incentive was given for participation. The research was approved and conducted according to the ethics committee No. 0111335 of “Università degli Studi dell’Insubria”, Varese, Como, Italy.

Pediatricians were asked to complete the questionnaire voluntarily, without any compensation or benefit. The questionnaire was specifically designed for this study. Before participating, all respondents provided informed consent and agreed to the privacy policy for the protection of personal data. No personally identifiable information was collected, and the data were analyzed only in aggregate form. The Google Form service was used to collect all responses anonymously. The resulting data file used for analysis was devoid of any identifiers such as email addresses, IP addresses, or other electronic identifiers.

This study adhered to the principles outlined in the Declaration of Helsinki, ensuring ethical considerations were followed.

### 2.2. Study Design

This study is a cross-sectional one.

The post hoc power of the study was calculated based on the main outcome of the method used to diagnose OSAS.

In the sample considered, 60.6% affirmed the use of PSG. In the literature, there are no studies that analyze this aspect in a very specific way between Italian pediatricians, so we considered a general percentage of 50%. To achieve 80% power with an alpha set at 0.05, the sample size was calculated at 194.

### 2.3. Questionnaire Variables

#### 2.3.1. Personal Details

- Sex.
- Provenance.
- Employment.
- In the past 5 years, have you attended a lecture/congress on OSAS about developmental age?
- Area.

#### 2.3.2. Diagnosis

- What premonitory NOCTURNAL OSAS symptoms do you predominantly identify in children?
- What premonitory diurnal OSAS symptoms do you identify predominantly in children?
- Do you consider WEIGHT among the predisposing factors of OSAS?
- What methods do you use most to diagnose OSAS?
- Do you have difficulty prescribing polysomnography to children?
- Does the first visit include a clinical history regarding sleep and snoring?
- Do you evaluate oral breathing during check-ups?
- During follow-up visits, do you dwell on the evaluation of the oral–otorhinolaryngology area?
- What are the anatomo-functional predisposing factors that you have found the most?

#### 2.3.3. Treatment General Knowledge

- Are you aware of the regional diagnostic and therapeutic care pathways (PDTA) for the prevention of OSAS in growing patients?
- Are you familiar with Continuous Positive Airway Pressure (CPAP) breathing support therapy?
- Are you familiar with the palatal expander?
- Do you think the use of the palatal expander is useful for OSAS in children?
- Do you think that tonsillectomy is essential to resolve OSAS?

#### 2.3.4. Multidisciplinary Approach

- Among these medical roles, who do you collaborate with?
- Whom do you refer OSAS patients to?

### 2.4. Statistical Analysis

The statistical analysis performed consisted of descriptive analysis, frequencies, percentages, Chi Square, and Logistic Regression.

The Pearson Chi Square test (two-tailed) was used to test possible correlations between “Provenence”, “Sex”, and “Employment”; “Provenence” and “In the past 5 years, have you attended a lecture/congress on OSAS about developmental age?”; “Provenence” and “Do you have difficulty prescribing polysomnography to children?”; “Provenence” and “Are you familiar with Continuous Positive Airway Pressure (CPAP) breathing support therapy?”; and “Sex” and “Employment?”. It used a Logistic Regression of “Sex” and “Provenence” as factors and “Employment” as a dependent variable to predict the probability of a particular “Employment” considering “Sex” and “Provenence”. The *p* and alpha values were set at 0.05.

## 3. Results

The sample consisted of 155 males (51.8%) and 142 females (47.5%), and 2 did not specify their sex. The survey was conducted exclusively in Italy, and the participants were divided based on their geographic origin: 76 pediatricians (25.4%) were from the north, 223 (74.6%) were from the Center/South of Italy; in particular, 119 (39.8%) were from the center, and 104 (34.8%) were from the south. The sample included 108 hospital pediatricians, 141 family pediatricians, and 50 self-employed pediatricians. Out of the total sample, only 172 pediatricians had attended a lecture or congress about pediatric OSAS in the last 5 years (Table 1).

Regarding the consideration of the oral and otorhinolaryngologist area during checkups, 206 pediatricians (68.9%) reported taking them into account. The most frequently identified nocturnal symptoms, in order of frequency, were snoring (68.9%), restless sleep (50.8%), sudden awakenings (47.8%), enuresis (46.8%), sleepwalking (42.1%), and awakening with a feeling of suffocation (33.1%) (Table 1).

The pediatricians reported being able to identify certain diurnal OSAS symptoms, including hyperactivity (64.2%), hypoactivity (51.5%), learning disorders (50.2%), neurocognitive disorders (43.5%), obesity (43.1%), growth delay (41.8%), oral breathing (38.8%), and headaches (33.1%) (Table 1).

The majority of the sample (70.6%) reported considering the weight of their patients, and 62.8% claimed to be aware of the regional diagnostic–therapeutic–assistance pathway. As a diagnostic method for OSAS, 197 pediatricians (65.9%) reported using questionnaires, while only 95 (31.8%) used hospital polysomnography, and 86 (28.8%) prescribed polysomnography for home use. Additionally, 233 pediatricians (77.9%) found it challenging to prescribe polysomnography for children and incorporate questions about sleep and snoring in the medical history (Table 1).

The most commonly found predisposing anatomical–functional factors, in order of frequency, were hypertrophy of adenoids and tonsils (57.2%), deformities (54.2%), prematurity (53.2%), cleft palate (40.1%), nasal polyps (38.1%), maxillary hypoplasia (37.5%), cerebral palsy (33.8%), and contracted palate (32.1%) (Table 1).

Furthermore, 129 pediatricians (43.1%) reported collaborating with pediatric dentists, 153 (51.2%) with otorhinolaryngologists, 144 (48.2%) with sleep doctors, 89 (29.8%) with general dentists, and 150 (50.2%) with orthodontists (Table 1).

Among the sample, 216 pediatricians (72.2%) know what a CPAP is, and 214 (71.6%) have knowledge about the palatal expander. Additionally, 212 (70.9%) affirm that this device is useful in treating OSAS in young patients (Table 1).

During checkups, 215 (71.9%) evaluate oral breathing, and 164 (54.8%) consider tonsillectomy to be important in the treatment of OSAS (Table 1).

We used a statistical analysis of “Sex”, “Provenance”, and “kind of employment”. They were tested for normality with the Shapiro–Wilk Test. There was not any significant statistical difference when comparing pediatricians from different areas of the country attending lectures or congress about OSAS in recent years. There was not any significant statistical difference in difficulty prescribing polysomnography when comparing pediatricians from different areas of the country. There were not any significant statistical sex differences in being familiar with CPAP when comparing pediatricians from different areas of the country. We found a significant statistical difference between “Sex” and “Employment?”; more female pediatricians are self-employed than males (*p* < 0.05) (Table 2). We used a Logistic Regression of “Sex” and “Provenence” as factors and “Employment” as a dependent variable to predict the probability of a particular “Employment” considering “Sex” and “Provenence” but did not find any statistically significant result.

## 4. Discussion

The aim of this study is to evaluate the level of knowledge among pediatricians in Italy regarding the diagnosis of pediatric OSAS. The analyzed sample is homogeneous; in fact, the percentage of females and males is very similar. Half of the sample reported attending a lecture or congress on OSAS in the last five years; this is a significant aspect considering that the majority of the sample comprises family and hospital pediatricians. This underscores the possibility of providing routine checkups for every child by doctors knowledgeable about this syndrome, even if a good percentage of the sample (42.5%) did not attend any lecture or congress, which means that there is a significant need to increase the awareness about this syndrome. Therefore, it is crucial to promote basic knowledge about this condition among pediatricians. Considering the sample, there was no difference between pediatricians from different parts of the country in terms of attending a lecture or congress on OSAS in the last five years. This study evidenced that there was no difference regarding “difficulty prescribing polysomnography to children” among different areas of the country, which is a good sign because it means that probably every patient might have access to this very important diagnostic examination. Polysomnography (PSG) is a commonly used diagnostic test in the evaluation of pediatric OSAS. PSG provides a comprehensive assessment of a child’s sleep patterns, breathing, and physiological functions during sleep. In the same way, there was no difference in “being familiar with CPAP”, which confirms a potential similar knowledge all over the nation. All these aspects can reassure patients from different areas of the country that they can have the same level of healthcare without the need to move towards different geographic areas, although it is remarkable that there is a significant part of the sample (27.2%) that needs to have basic knowledge about this important therapeutic instrument, which is still considered the gold standard of OSAS therapy [20].

Regarding the survey results, most of the sample affirmed that they consider the oral and otorhinolaryngology aspects when evaluating OSAS. This is remarkable considering that OSAS is a multifactorial disease, but it is still noticeable that part of the sample does not consider these areas (31.1%). These parts have significant importance in predicting the possible presence of apnea because they are strictly connected with the posterior airway space, PAS, which can have a significant influence on the disease. PAS is very connected with oral and facial deformities that should be recognized in the first pediatric checkup. Some of the pediatricians in the sample affirm being aware of deformities as another set of signs and symptoms. Deformities such as a retro-positioned mandible or undergrown lower jaw can cause a significant reduction in the posterior airway space (PAS), increasing the likelihood of OSAS and its associated symptoms. Furthermore, a contracted palate is another aspect that deserves attention since rapid palate expansion can help reduce oral breathing and increase the dimensions of the upper respiratory airways [13,21,22]. In some cases, there is a compelling need for pediatricians to refer their patients to dentists, maxillofacial surgeons, and otorhinolaryngologists, but that can happen only if they have a basic knowledge of these anatomical areas.

A significant portion of the sample recognized the importance of adenotonsillectomy, which is a complex part of the OSAS diagnosis. The literature identifies adenotonsillectomy as the primary treatment for OSAS, but its effectiveness and ability to provide a definitive solution are uncertain. Nowadays, adenoidectomy is not always performed with tonsillectomy. Instead, tonsillectomy is highly recommended in young patients, particularly when there is hypertrophy and a certain number of episodes of infection, as it can improve various conditions, including OSAS [14,23,24,25]. 

However, each case should be evaluated carefully to ensure the necessity and appropriateness of surgery. In this study, the positive aspect is the good average knowledge among pediatricians about the importance of adenoids and tonsils, with the potential for further improvement through increased awareness [21].

This survey also sheds light on the common symptoms that healthcare professionals use to identify OSAS in their patients. The most prevalent nocturnal symptoms identified are snoring, restless sleep, sudden awakenings, enuresis, and sleepwalking. The most common daytime symptoms identified were hyperactivity, hypoactivity, learning disorders, neurocognitive disorders, and obesity. The most recognized symptom by pediatricians is snoring, which can also occur independently of OSAS. Approximately 10% of children snore regularly, and up to 5% of children have OSAS, according to the literature [4]. In this way, snoring can be a premonitory symptom that has to be considered, but it cannot be the only one; healthcare providers should take into account other signs and symptoms. OSAS can result in various health problems, including behavioral and cognitive issues, cardiovascular problems, and poor growth. Restless sleep is the second most recognized symptom by the sample, and enuresis is the third, which is known to decrease in patients who have undergone adenoidectomy and tonsillectomy [24]. Enuresis can be a factor to consider in patients with a possible diagnosis of OSAS. Several studies have demonstrated a strong correlation between enuresis and OSAS in children, and a study proved that children with enuresis can also have OSAS [7,25].

Sleepwalking was also considered in the questionnaire, although the evidence linking it to OSAS is not very strong. Furthermore, the pediatricians reported paying close attention to the association between weight and the analyzed disease, emphasizing its significance in OSAS. 

The prevalence of OSAS is significantly higher in obese individuals compared to those of normal weight [26].

The combination of obesity and OSAS poses a higher cardiovascular risk, as both conditions independently contribute to the development of hypertension, insulin resistance, dyslipidemia, and systemic inflammation [27].

In pediatric OSAS, oral breathing plays a significant role and influences the severity and management of the condition [28]. 

The significance of oral breathing in pediatric OSAS lies in its association with disease severity. Studies have shown that children who predominantly breathe through their mouth during sleep tend to have more severe OSAS compared to those who primarily breathe through their nose [28]. Oral breathing exacerbates upper airway collapsibility, leading to increased resistance, greater sleep fragmentation, and decreased oxygen saturation levels during episodes of apnea and hypopnea [29]. Oral breathing is a common health issue that can lead to malocclusion and improper tongue posture, causing palate contraction, increased overjet, and altered craniofacial growth. Narrow upper jaws can contribute to nasal obstruction, making nasal breathing difficult, and individuals may compensate by breathing through their mouth chronically. The use of a palatal expander can address the underlying skeletal issue of a narrow upper jaw, potentially improving nasal airflow and encouraging nasal breathing. 

Studies demonstrated that rapid maxillary expansion significantly improved nasal airflow and reduced oral breathing. According to the outcomes of this article, the majority of the sample (71.6%) had an awareness of the palatal expander and the implications of unhealthy oral breathing [30,31].

These factors contribute to the overall disease burden and impact the child’s quality of life. It is very important to treat oral breathing as early as possible to change this pathological pathway, and pediatricians are usually the first physicians who check young patients. In the sample, 27.8% do not consider oral breathing in their routine checks. For this reason, a better educational program for pediatricians to learn the importance of this dysfunction is still needed. It is reasonable to affirm that the knowledge that pediatricians can have about this theme depends on the collaboration with other specialists, in particular, otorhinolaryngologists and dentists. In fact, only 53.5% and 47.8% affirm to cooperating with otolaryngologists and general dentists. Collaboration with dentists, particularly orthodontists, is crucial because OSAS is recognized as a multifactorial illness. In some cases, the involvement of otorhinolaryngologists and dentists is essential in resolving this health issue. Dentists in Italy should play a role in the early recognition of this disease and work collaboratively with other medical professionals to address it effectively.

However, nowadays, there is also the possibility of treating mild and moderate OSAS using mandibular advancement devices (MAD). In this case, dentists play a significant role as they can prescribe and provide patients with these devices [32]. 

The Italian pediatric diagnostic therapeutic pathway for pediatric Obstructive Sleep Apnea Syndrome (OSAS) is not well-documented in the literature. However, considering how this disease can impact the quality of life for patients, it is crucial for pediatricians to have clear guidelines to follow. It is noteworthy that half of the sample population has knowledge about the regional diagnostic and therapeutic guidelines, which is an important result. The diagnostic process consists of two phases: the first phase involves diagnostic suspicion based on the use of questionnaires, while the second phase includes a checkup with a clinical expert in sleep disorders. Based on the results, there is a solid foundation that can be improved by spreading awareness and knowledge about these diagnostic phases among pediatricians [33].

These findings emphasize the need for ongoing education and training initiatives, comprehensive evaluation of premonitory symptoms, the utilization of validated assessment tools, and collaborative care to ensure the effective diagnosis and management of pediatric OSAS. It can be useful to analyze, in the future, the possible role that artificial intelligence can have in the diagnosis and treatment of OSAS [34,35].

### Study Limitations

Overall, it is important to mention some limitations of this study. All the limits of an anonymous questionnaire were considered. By its very nature, a questionnaire involves personal perceptions, personal knowledge, and opinions on a certain subject. Some people might not be aware of the significance of some questions or the intended meaning of some answers. This study has limitations caused by the complexity of the pathology.

According to the Checklist for Reporting Results of Internet E-Surveys (CHERRIES), the result of a web survey using an anonymous questionnaire cannot be evaluated like a certain result, but it can only be considered a hypothesis that should be validated in a more thoroughly checked study. However, this study cannot be considered a web survey because it was widespread through clinical practices. Another weakness of this survey is the absence of the sample subjects’ age. The questionnaire was not validated, even if it included an important sample. The absence of validation reduces the value of the results, but this manuscript can be considered a pilot of significant importance for further studies.

## 5. Conclusions

According to the results of this manuscript, there is evidence of a good level of knowledge among pediatricians about OSAS (Obstructive Sleep Apnea Syndrome), oral breathing, and the necessity of a multidisciplinary approach to this syndrome. This last aspect is very important for pediatricians because they are one of the first lines of health checkups for the majority of young patients and can refer them to other clinicians. In fact, 43.1% of the sample reported collaborating with pediatric dentists, 51.2% with otorhinolaryngologists, 48.2% with sleep doctors, 29.8% with general dentists, and 50.2% with orthodontists. This is an important result that can even be improved if the right awareness is widespread. This study indicates a mild diffusion of basic knowledge about respiratory problems; in this way, 206 pediatricians (68.9%) reported taking into account the oral and otorhinolaryngologist area during checkups. In the future, disseminating more information about OSAS and all the health issues associated with this syndrome is suggested, considering the impact it can have. This approach will effectively promote a standard clinical approach to diagnosing OSAS throughout the country. Further research is needed to expand our understanding of regional variations and optimize treatment strategies for the kind of population considered.

## Figures and Tables

**Table 1:** Questionnaire in English language.

	Frequencies	Percentages
Sex		
Female	142	47.5
Male	155	51.8
Chose not to specify	2	0.7
Provenance		
North Italy	76	25.4
Central/South Italy	223	74.6
	Employment	
Self-employment	50	16.7
Pediatrician	141	47.2
Hospital worker	108	3.1
In the past 5 years, have you attended a lecture/congress on OSAS about developmental age?		
YES	172	57.5
NO	127	42.5
During follow-up visits, do you dwell on the evaluation of the oral–otorhinolaryngology area?		
SI	206	68.9
NO	93	31.1
What premonitory NOCTURNAL OSAS symptoms do you predominantly identify in children?		
Snoring	206	68.9
Restless sleep	152	50.8
Sudden awakenings	143	47.8
Enuresis	140	46.8
Sleepwalking	126	42.1
Awakenings with feelings of suffocation	99	33.1
What premonitory DIURNAL OSAS symptoms do you identify predominantly in children?		
Hyperactivity	192	64.2
Hypoactivity	154	51.5
Learning disorders	150	50.2
Neurocognitive disorders	130	43.5
Obesity	126	42.1
Growth retardation	125	41.8
Oral breathing	116	38.8
Headache	99	33.1
Do you consider WEIGHT among the predisposing factors of OSAS?		
Yes	211	70.6
NO	88	29.4
Are you aware of regional PDTAs for the prevention of OSAS in growing patients?		
YES	188	62.8
NO	110	36.8
Did not respond	1	0.3
What methods do you use most to diagnose OSAS?		
Questionnaire	197	65.9
Laboratory polysomnography	95	31.8
Home polysomnography	86	28.8
Do you have difficulty prescribing polysomnography to children?		
YES	233	77.9
NO	65	21.7
Did not respond	1	0.3
Does the first visit include a clinical history regarding sleep and snoring?		
YES	233	77.9
NO	66	221
What are the anatomo-functional predisposing factors that you have found the most?		
Adenotonsillar hypertrophy	171	57.2
Malformations	162	54.2
Prematurity	159	53.2
Cleft palate	120	40.1
Nasal polyps	114	38.1
Maxillary hypoplasia	112	37.5
Cerebral palsy	101	33.8
Contracted palate	96	32.1
Among these medical roles, whom do you collaborate with?		
Pediatric dentist	173	57.9
Otolaryngologist	160	53.5
Sleep doctor	149	49.8
General dentist	143	47.8
Orthodontist	118	39.5
Are you familiar with Continuous Positive Airway Pressure (CPAP) breathing support therapy?		
YES	216	72.2
NO	82	27.4
Did not respond	1	0.3
Are you familiar with the palatal expander?		
YES	214	71.6
NO	85	28.4
Do you think the use of the palatal expander is useful for OSAS in children?		
YES	212	70.9
NO	84	28.1
Did not respond	1	0.3
Do you evaluate oral breathing during check-ups?		
YES	215	71.9
NO	83	27.8
Did not respond	1	0.3
Whom do you refer OSAS patients to?		
Otolaryngologist	153	51.2
Orthodontist	150	50.2
Pediatric dentist	129	43.1
Sleep doctor	144	48.2
General dentist	89	29.8
Do you think that tonsillectomy is essential to resolve OSAS?		
YES	164	54.8
NO	130	43.5
Did not respond	5	1.7

**Table 2:** Statistical analysis.

	Value	Significance
Pearson Chi Square test: “Where do you live?” and “In the last 5 years have you attended a lecture/congress on OSAS in developmental age?	X2(1) = 0.006	*p* = 0.940
Pearson Chi Square test: “Where do you live?” and “Do you have difficulty prescribing polysomnography to children?”	X2(1) = 0.377	*p* = 0.828
Pearson Chi Square test: “Where do you live?” and “Are you familiar with Continuous Positive Airway Pressure Respiratory Support Therapy (CPAP)?”	X2(1) = 0.416	*p* = 0.812
Pearson Chi Square test: “Sex” and “Where do you work?”	X2(1) = 6.502	*p* = 0.039

## Data Availability

The raw data supporting the conclusions of this article will be made available by the authors on request due to privacy.
